# Divergent immune priming responses across flour beetle life stages and populations

**DOI:** 10.1002/ece3.2532

**Published:** 2016-10-09

**Authors:** Imroze Khan, Arun Prakash, Deepa Agashe

**Affiliations:** ^1^ National Centre for Biological Sciences Tata Institute of Fundamental Research Bangalore India

**Keywords:** ontogenic immune priming, transgenerational immune priming, *Tribolium castaneum*, variability, wild populations, within‐generation immune priming

## Abstract

Growing evidence shows that low doses of pathogens may prime the immune response in many insects, conferring subsequent protection against infection in the same developmental stage (within‐life stage priming), across life stages (ontogenic priming), or to offspring (transgenerational priming). Recent work also suggests that immune priming is a costly response. Thus, depending on host and pathogen ecology and evolutionary history, tradeoffs with other fitness components may constrain the evolution of priming. However, the relative impacts of priming at different life stages and across natural populations remain unknown. We quantified immune priming responses of 10 natural populations of the red flour beetle *Tribolium castaneum*, primed and infected with the natural insect pathogen *Bacillus thuringiensis*. We found that priming responses were highly variable both across life stages and populations, ranging from no detectable response to a 13‐fold survival benefit. Comparing across stages, we found that ontogenic immune priming at the larval stage conferred maximum protection against infection. Finally, we found that various forms of priming showed sex‐specific associations that may represent tradeoffs or shared mechanisms. These results indicate the importance of sex‐, life stage‐, and population‐specific selective pressures that can cause substantial divergence in priming responses even within a species. Our work highlights the necessity of further work to understand the mechanistic basis of this variability.

## 1 Introduction

Growing evidence suggests that a low dose of a pathogen may prime the immune response in insects, reducing the risk and severity of infection by the same pathogen later in life. Evidence for such priming‐induced immune protection has been reported in many insects including mealworm beetles (Daukšte, Kivleniece, Krama, Rantala, & Krams, 2012), bumble bees (Sadd & Schmid‐Hempel, 2006; Tidbury, Pedersen, & Boots, 2011), silkworms (Miyashita, Kizaki, Kawasaki, Sekimizu, & Kaito, 2014), fruit flies (Pham, Dionne, Shirasu‐Hiza, & Schneider, 2007), mosquitoes (Contreras‐Garduño, Rodríguez, Rodríguez, Alvarado‐Delgado, & Lanz‐Mendoza, 2014), and flour beetles (Roth, Sadd, Schmid‐Hempel, & Kurtz, 2009). Immune priming can also confer sustained protection via (i) ontogenic priming, where the benefit of priming can persist through metamorphosis (Moreno‐García, Vargas, Ramírez‐Bello, Hernández‐Martínez, & Lanz‐Mendoza, 2015; Thomas & Rudolf, 2010) and (ii) transgenerational immune priming, where the benefits are manifested in the next generation (Dubuffet et al., 2015; Moreau, Martinaud, Troussard, Zanchi, & Moret, 2012; Sadd & Schmid‐Hempel, 2006; Sadd & Schmid‐hempel, 2009; Zanchi, Troussard, Moreau, & Moret, 2012). Theoretical models show that within‐generation and transgenerational immune priming can significantly alter pathogen persistence (Tidbury, Best, & Boots, 2012) and reduce infection intensity in populations (Tate & Rudolf, 2012). Thus, it is clear that immunological memory is widespread in insects, and immune priming may have large impacts on the outcome of host–pathogen interactions.

Although we have begun to understand immune priming in many insects, it is not clear how priming evolves. This is partly because the strength, consistency, and relevance of immune priming in natural populations remains largely unexplored and is difficult to gauge from laboratory studies. Other aspects of immune function (postinfection survival and encapsulation ability) vary across fruit fly populations (Corby‐Harris & Promislow, 2008; Kraaijeveld & Van Alphen, 1995), and parasite burden is strongly correlated with the strength of the innate immune response across damselfly populations (Kaunisto & Suhonen, 2013). Similarly, immune priming responses may also vary across natural populations. In laboratory populations, immune priming is affected by the presence of other pathogens (Sadd & Schmid‐hempel, 2009) and food availability (Freitak, Heckel, & Vogel, 2009). However, the impact of these factors on immune priming in natural populations is unknown. Wild populations likely face substantial spatial and temporal variation in pathogen diversity, pathogen abundance, and resource availability, generating variability in the strength of selection on immune priming. Priming also imposes fitness costs in some laboratory populations (Contreras‐Garduño et al., 2014), potentially generating tradeoffs with other immune responses, or between different types of immune priming. Finally, these fitness costs may also vary as a function of sex and developmental stage. For instance, life‐history theory predicts that females should generally evolve higher immune competence than males (Nunn, Lindenfors, Pursall, & Rolff, 2009; Rolff, 2002); hence, males may gain more benefits from priming than females (Moreno‐García et al., 2015). Similarly, variable costs of infection across life stages are also predicted to select for stronger priming responses at specific developmental stages (Tate & Rudolf, 2012). A detailed analysis of such variability can indicate factors that influence the evolution of immune priming. Unfortunately, very few studies have quantified priming in wild insect populations (but see (Reber & Chapuisat, 2012) (ants), (Gonzalez‐Tokman, Gonzalez‐Santoyo, Lanz‐Mendoza, & Cordoba Aguilar, 2010) (damselflies), and (Tate & Graham, 2015) (closely related flour beetle species)), and none have measured variation in priming responses across multiple natural populations.

**Figure 1:**
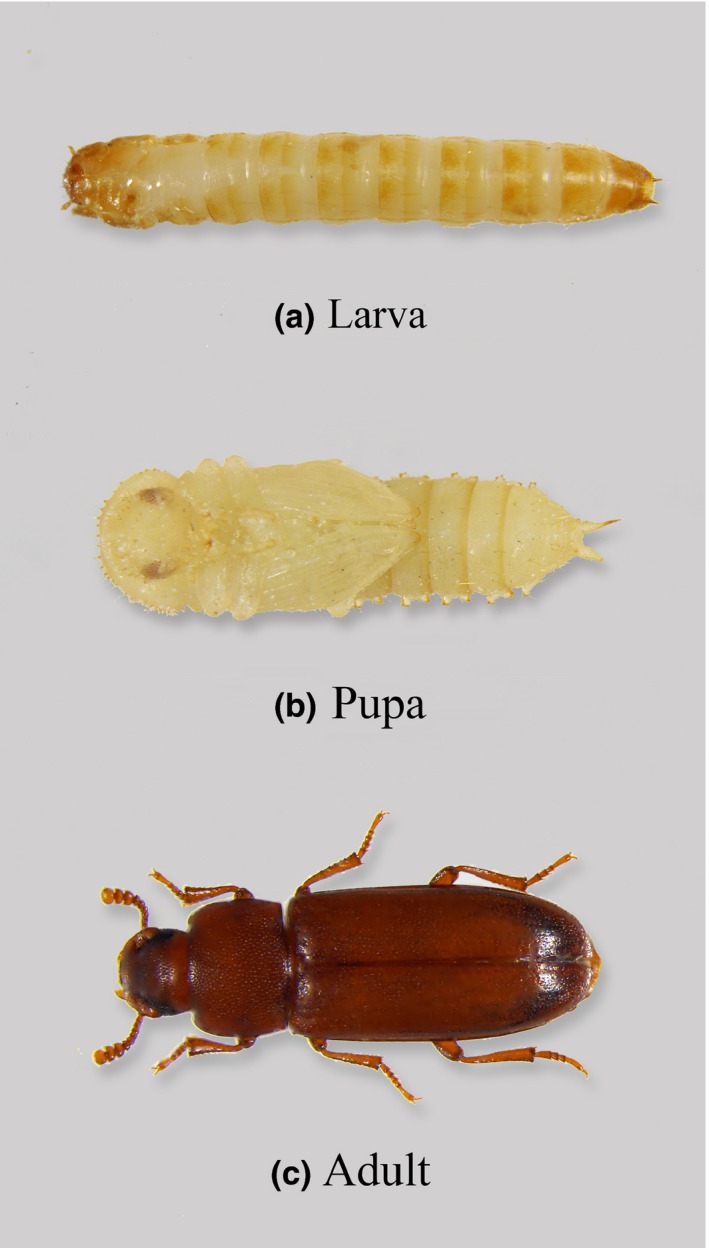
Different life stages of red flour beetle *Tribolium castaneum*

We systematically analyzed immune priming responses of 10 populations of the red flour beetle *Tribolium castaneum* (Figure 1) collected from different locations across India (see Fig. S1 for collection sites). In the laboratory, flour beetles show within‐life stage (WLS) (Roth et al., 2009), ontogenic (ONT) (Thomas & Rudolf, 2010), and transgenerational (TG) immune priming (Roth et al., 2010), making them an ideal model system to understand the occurrence and abundance of these different types of immune priming responses. We addressed three major questions: (i) Does the immune priming response vary across natural populations and as a function of sex and life stage? (ii) Are the different types of priming responses equally beneficial? (iii) Are the different types of immune priming responses correlated? We found that ontogenic immune priming provided greater protection against re‐infection, compared with within‐life stage or transgenerational priming. Finally, our data reveal novel sex‐specific links between various forms of immune priming, perhaps representing tradeoffs or a shared mechanistic basis. Our work is the first report of large within‐species variability of priming response across sexes and life stages in natural insect populations. We hope that our results motivate further investigations to confirm and understand the ecological, evolutionary, and mechanistic basis of the observed variability and associations between priming at different stages.

## 2 Methods

### 2.1 Beetle collection and experimental individuals

Although immune priming responses should be measured on individuals directly collected from the wild (i.e., grain warehouses), this is difficult to do for the following reasons. First, natural beetle populations do not always have enough individuals of different stages to allow sufficient replication. Second, it is impossible to account for the many factors that may increase within‐population variability in immune responses, such as individual age, migration, and dietary history, and immediate local environment. Controlling for within‐population variability in immune priming is essential to quantify variability between populations, which was the major goal of our study. Hence, we established large laboratory populations using wild‐collected beetles (maintaining most of the initial genetic variability) and then quantified the immune priming response of individuals of the same age reared under identical conditions. We collected 50–100 *T. castaneum* adults from a grain warehouse in each of nine cities across India. Of the 10 populations analyzed here, eight were from different cities and two were collected from different warehouses in a single city (Fig. S1). We allowed all adults from a site to oviposit for a week on whole‐wheat flour at 34°C to start a large laboratory population (>2,000 individuals). We maintained these stock populations on a 45‐day discrete generation cycle for 9–10 generations before starting experiments.

To generate experimental individuals of equivalent age from all populations, we allowed ~1,000 adults from each population to oviposit in 350 g wheat flour for 48 hr. We removed the adults and allowed offspring to develop for ~3 weeks until pupation, collecting pupae daily after this period. We housed 3–4 pupae of each sex separately in 2‐ml microcentrifuge tubes containing 1 g flour for 2 weeks. As pupae typically eclose in 3–4 days, we obtained ~11‐day‐old sexually mature virgin adults for immune priming experiments. For experiments with larvae, we allowed adults to oviposit in 350 g flour for 24 hr and collected larvae after 10 days (eggs hatch in 2–3 days; thus, experimental larvae were ~8 days old). In a separate experiment, we found that eggs from all populations developed at a similar rate (Fig. S2), confirming that we tested all populations at equivalent developmental stages.

### 2.2 Immune priming and challenge

For each type of immune priming, we tested all populations together to allow a direct comparison across populations. However, given logistical constraints, we had to test males and females in separate blocks. Note that we only measured maternal TG immune priming in our experiments and did not measure paternal TG priming. For all infections, we used a strain of *Bacillus thuringiensis* (DSM. No. 2046). Originally isolated from a Mediterranean flour moth, this is a natural insect pathogen that imposes significant mortality in flour beetles (Abdel‐Razek, Salama, White, & Morris, 1999). On the evening before priming, we inoculated 10 ml nutrient broth (Difco) with cells from a −80°C stock of *B. thuringiensis*. We incubated the growing culture overnight in a shaker at 30°C until it reached an optical density of 0.95 (measured at 600 nm in a Metertech UV/vis spectrophotometer, SP8001). We centrifuged the culture at 2823 g for 10 min, removed the supernatant, and resuspended the pellet in 100 μl insect Ringer solution (7.5 g NaCl, 0.35 g KCl, 0.21 g CaCl_2_ per liter) to make bacterial slurry. We killed the bacteria in a heat block at 90°C for 20 min as described earlier (Khan, Prakash, & Agashe, 2015; Roth et al., 2009). We used heat‐killed bacteria to prime individuals, as this would elicit an immune response without a direct cost of infection.

**Figure 2:**
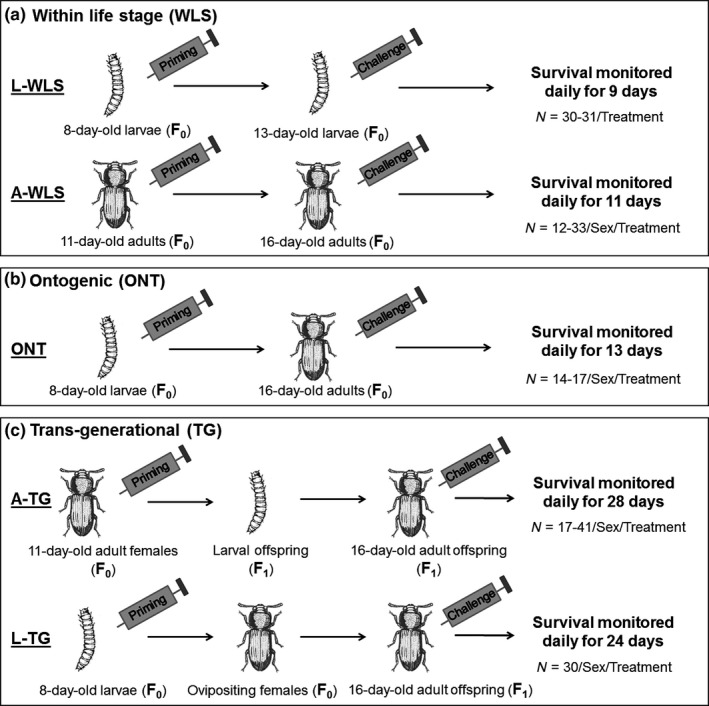
Experimental design to measure the strength of immune priming responses at different stages: (a) Within‐life stage priming (individuals primed and challenged as larvae (L‐WLS) or adults [A‐WLS]), (b) ontogenic priming (individuals primed as larvae and challenged as adults), and (c) transgenerational maternal priming (females primed as larvae [L‐TG] or adults [A‐TG] were paired with uninfected virgin males and their offspring were challenged). Sample sizes of challenged individuals are provided for each treatment (priming and control) and sex

To prime individuals, we pricked them with a 0.1‐mm minutien pin (Fine Science Tools, Fosters City, CA, USA) dipped either in heat‐killed bacteria (primed) or in sterile insect Ringer solution (mock priming control). To minimize damage to internal organs, we pricked individuals laterally between the head and thorax (adults) or between the last two segments (larvae). After priming (or mock priming), we isolated individuals in wells of 96‐well microplates containing flour. For subsequent immune challenge, we pricked individuals as described above, but used live bacterial slurry (without heat‐killing). This procedure delivers ~8 × 10^3^ live bacterial cells to each individual. We found negligible mortality after priming and before challenge treatment (comparison of the number of primed and challenged individuals in Table S1). Below, we describe the timeline and number of replicate challenged individuals for each immune priming assay (also see Figure 2).

1. Within‐life stage immune priming in larvae (L‐WLS) (*n* = 30–31/treatment/population) and adults (A‐WLS) (females: *n* = 32–33/treatment/population; males: *n* = 12/treatment/population): We primed 11‐day‐old adults or 8‐day‐old larvae as described above and then isolated them in wells of 96 well microplates containing wheat flour (25 mg per well) for 5 days. After the immune challenge, we returned them to wells of fresh 96‐well microplates containing flour. We recorded individual survival every 6 hr for the first two days and then daily around 10 pm for the following 7 and 9 days for larvae and adults, respectively.
2. Ontogenic (ONT) immune priming (females: *n* = 15–17/treatment/population; males: *n* = 14–17/treatment/population): We primed 8‐day‐old larvae, isolated them as described above (for WLS), and sexed them at the pupal stage. On day 16 postemergence, we challenged each adult and then noted its survival every 6 hours for 48 hr postinfection and then daily around 10 pm for the next 11 days.
3. Transgenerational benefits of priming adult females (A‐TG) (female offspring: *n* = 17–41/treatment/population; male offspring: *n* = 21–26/treatment/population): We primed 11‐day‐old virgin adult females (*n* = 28–35/treatment/population), and the next day we allowed each female to mate with an uninfected, 11‐day‐old virgin male for 2 days in a 1.5‐ml microcentrifuge tube containing 1 g wheat flour. We then separated the females and allowed them to oviposit for 24 hr in a 60‐mm plastic petriplate containing 5 g wheat flour. We incubated offspring from each female in the respective oviposition plates. Three weeks later, we counted pupae from each oviposition plate and found that females produced an average of 10–12 pupae (Figs. S3–S6). To exclude females whose fitness was significantly lower than average, we excluded plates with <5 pupae. We pooled pupae from all other oviposition plates, sexed them, and randomly distributed them into wells of 96‐well microplates containing flour. Note that this exclusion did not skew the offspring pool in favor of the most fecund females (Figs. S3–S6). On day 16 postemergence, we challenged adult offspring as described earlier. We recorded their survival every 6 hr for 2 days and then daily around 10 pm until all of them died.
4. Transgenerational benefits of priming females at larval stage (L‐TG) (female offspring: *n* = 30/treatment/population; male offspring: *n* = 30/treatment/population): We primed 8‐day‐old larvae and isolated them in wells of 96‐well microplates until pupation. We sexed and redistributed them in fresh 96‐well plates. On day 10 postemergence, we paired each adult female (*n* = 29–35/treatment/population) with an uninfected virgin male (11‐day‐old) to constitute mating pairs. We discarded the males after 48 hr, and allowed females to oviposit for 24 hr in 60 mm Petriplates with 5 g of wheat flour. Three weeks later, we collected adult offspring and challenged them on day 16 postemergence with live bacteria as described above for A‐TG. We noted beetle mortality every 6 hr for 2 days and then daily around 10 pm until all of them died.

### 2.3 Data analysis

We analyzed postinfection survival data for each population, sex and immune priming type separately using Cox proportional hazard survival analysis with priming treatment as a fixed factor (see Figs. S7–S15 for survival curves). We noted individuals that were still alive at the end of the experiment as censored values. We calculated the strength of a given type of immune priming response within each population (and sex) as the estimated hazard ratio of unprimed versus primed groups (hazard ratio = rate of deaths occurring in unprimed group/rate of deaths occurring in primed group). A hazard ratio significantly greater than one indicates a greater risk of death after infection in the unprimed (control) compared with primed individuals.

We used a nonparametric Wilcoxon rank sum test to analyze hazard ratios to test each of the following effects: (i) hazard ratios as a function of sex within each type of immune priming, (ii) hazard ratios as a function of life stage at priming (larvae vs. adults), and (C) hazard ratios as a function of different types of immune priming. We used a Steel–Dwass test to estimate pairwise differences, accounting for multiple comparisons.

We also wanted to test whether the strength of immune priming responses was correlated across types of priming. However, several populations did not show a significant immune priming response; hence, we could not use a linear regression approach. Therefore, we generated a contingency table, categorizing each population according to the occurrence (proportional hazard test: *p* < .05) or absence (proportional hazard test: *p* > .05) of each type of priming response (also see Figs. S7–S15). We then used a Fisher's exact test to determine whether the presence of the two types of immune priming was qualitatively associated across populations.

## 3 Results

### 3.1 The immune priming response varies across populations

**Figure 3:**
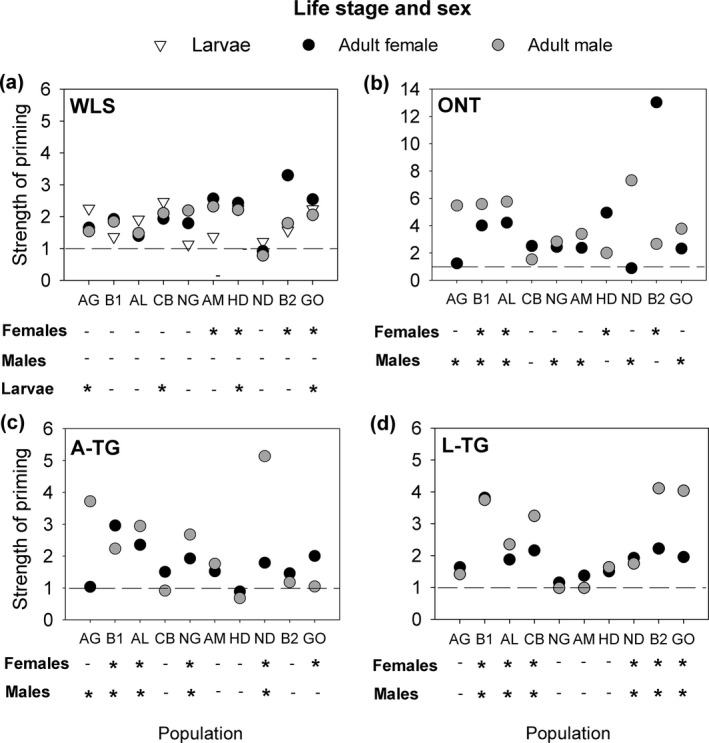
Variation in priming response across sexes, life stages, and populations. (a) Within‐life stage immune priming (WLS) benefit in larvae and adults, (b) ontogenic (ONT) immune priming benefit, (c) transgenerational benefits of priming adult females (A‐TG), (d) transgenerational benefits of priming females at larval stage (L‐TG). Strength of immune priming response was calculated as the hazard ratio of the proportion of deaths occurring in the unprimed group compared with the primed group under a proportional hazard model. Horizontal dashed lines in each panel indicate a hazard ratio of 1. “*”and “‐” denote significant (*p* ≤ .05) and nonsignificant (*p* > .05) impact of immune priming in each stage, sex, and population. Sample sizes for each group are given in Figure 2

We estimated the strength of immune priming as the proportional hazard ratio of individuals mock‐primed with sterile Ringer solution vs. primed with a pathogen (heat‐killed *B. thuringiensis*), followed by a subsequent infection with live *B. thuringiensis*. Surprisingly, we found that only about half the populations showed significant priming at a given stage, although all populations were capable of mounting multiple forms of immune priming (Figure 3). The immune priming response varied substantially in larvae as well as adult males and females across natural populations (Figure 3; Figs. S7–S15). We found that only a few populations showed significant within‐life stage immune priming as larvae (L‐WLS, 4/10 populations) or as adults (only females; A‐WLS, 4/10 populations) (Figure 3a). In contrast, at least one sex of many populations showed significant ontogenic (ONT, 9/10 populations; Figure 3b) and transgenerational benefits of priming adult females (A‐TG, 6/10 populations; Figure 3c). Our data also demonstrate long ranging impacts of transgenerational immune priming in several populations, whereby priming larvae improved postinfection survival of their adult offspring (L‐TG, 6/10 populations; Figure 3d). Finally, we found that populations B1 and B2 showed very different priming responses (Figure 3), although they were collected from different warehouses in the same city. Hence, geographical proximity does not seem to be a good predictor of similarity in immune responses.

We note that in the A‐TG priming assay for female offspring, the number of replicates varied substantially across treatments and populations (See Table S1; e.g., population B1: primed = 17 beetles, unprimed = 25 beetles; population AG: primed = 33 beetles, unprimed = 41 beetles). We failed to collect same number of pupae for each population and treatment, as some pupae eclosed earlier than expected. Hence, we tested the impact of lower sample size on the estimated hazard ratio using a bootstrap analysis (drawing *n* = 17 samples randomly with replacement, five subsamples per population). We found that the estimated hazard ratio was comparable for the lower vs. actual sample size (Fig. S16A). Next, we chose two populations where females either showed (e.g., GO) or lacked the priming response (e.g., AG), and performed another bootstrap analysis (*n* = 15–30 replicates/treatment/population) to estimate the impact of sample size on estimated hazard ratios. In each case, we found hazard ratios comparable to those estimated with the full data set (Fig. S16B, C), suggesting that different replicate sizes across treatments and populations did not have a large impact on hazard ratio estimates.

### 3.2 Effect of sex on immune priming

As explained in the methods, we tested the priming response of each sex separately. Hence, we could not directly test for an impact of sex in each population. Combining hazard ratios across populations, we did not find a consistent impact of sex on the strength of the immune priming response for any type of priming (Table 1a). However, in many populations, only one sex showed a significant priming response. For instance, the adult WLS response appears to be female‐limited, with males showing no priming in any population (Figure 3a). Similarly, in most populations that showed ontogenic priming, priming was beneficial for only one sex (7/9 populations; Figure 3b). However, unlike WLS, we did not find a systematic benefit of ONT priming: the sex that benefited from ONT priming varied across populations. We also did not find clear sex‐specific benefits of TG priming for offspring. We observed a benefit of A‐TG priming in offspring of both sexes (four populations) or only one sex (two populations) (Figure 3c). Intriguingly, all six populations with significant L‐TG priming showed a response in offspring of both sexes (Figure 3d). Thus, both males and females tend to show parallel benefits of L‐TG priming across populations. Overall, our results show that the impact of sex on immune priming varies both across populations and type of immune priming.

**Table 1:** Summary of Wilcoxon rank sum tests for the impact of (a) sex on hazard ratios within each type of immune priming, (b) life stage at priming on hazard ratios, and (c) types of immune priming on hazard ratios

Experiment	Type of priming	*df*	χ^2^	*p*
(a) Impact of sex	A‐WLS	1	0.365	.545
	ONT	1	1.12	.289
	A‐TG	1	0.205	.65
	L‐TG	1	0.28	.596
(b) Impact of life stage at priming		1	4.055	.04
(c) Impact of type of priming		4	16.13	.002

A‐WLS, within‐life stage immune priming in adults; ONT, ontogenic immune priming; A‐TG, transgenerational benefits of priming adult females, L‐TG, transgenerational benefits of priming females at larval stage.

### 3.3 Larval ontogenic priming maximizes protection against subsequent infection

**Figure 4:**
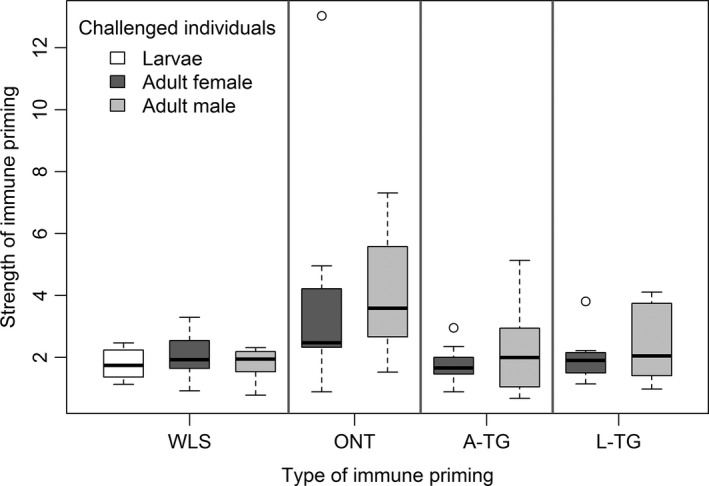
Strength of each type of immune priming response across different life stages and sexes. Strength of priming was calculated as described in Figure 3. Sample sizes for each assay are shown in Figure 2. WLS, within‐life stage immune priming, ONT, ontogenic priming; A‐TG, transgenerational benefits of priming adult females (A‐TG); L‐TG, transgenerational benefits of priming females at larval stage

Next, we tested the impact of priming life stage on the strength of the priming response. We found that priming at the larval stage was more beneficial and produced a greater response than priming adults (Table 1b). However, this result was driven primarily by larval ONT priming, which maximized postinfection survival in adults across priming types relative to the respective unprimed controls (Figure 4, Table 1c). Larval priming resulted in a ~threefold ONT survival benefit, compared with the twofold benefit observed for other forms of priming, including L‐WLS and L‐TG priming (Figure 4). We also found that across populations, the strength of ONT priming in females was more variable compared with WLS, L‐TG, or A‐TG priming (Bartlett's test for homogeneity of variance, *p* < .02 for each pairwise comparison; boxplots in Figure 4). For males, ONT priming was significantly more variable than WLS priming, but not other forms of priming. Together, our results suggest that among different types of immune priming, ONT priming responses are strongest and most variable.

We note that our finding of higher across‐population variability in ONT priming may also arise from the relatively low sample size per sex and population, compared with other forms of priming (see above). Our estimates of ONT priming may thus be relatively less precise for each population, potentially increasing between‐population variability despite low actual divergence between populations. Furthermore, primed females of population B2 showed a 13‐fold higher survival benefit that is substantially higher than other populations. To test whether population B2 overly influenced the relative benefit and variability of ONT priming, we reanalyzed hazard ratios after excluding females of population B2. We found that ONT priming still conferred a significantly higher survival benefit (Steel–Dwass test: *p* < .035 for each pairwise comparison) and more variable response (Bartlett's test for homogeneity of variance: *p* < .04 for each pairwise comparison) than WLS and A‐TG, but not L‐TG. Finally, we tested the repeatability of the ONT priming response for five of our populations (*n* = 12/sex/population), including population B2. The repeated priming assay with both sexes confirmed the pattern of variation of hazard ratios observed earlier, with highly correlated responses across both assays (see Fig. S17). Primed females of population B2 showed ~ninefold survival advantage of ONT priming, confirming their ability to produce a strong ONT response.

### 3.4 Associations between within‐generation and transgenerational immune priming

**Figure 5:**
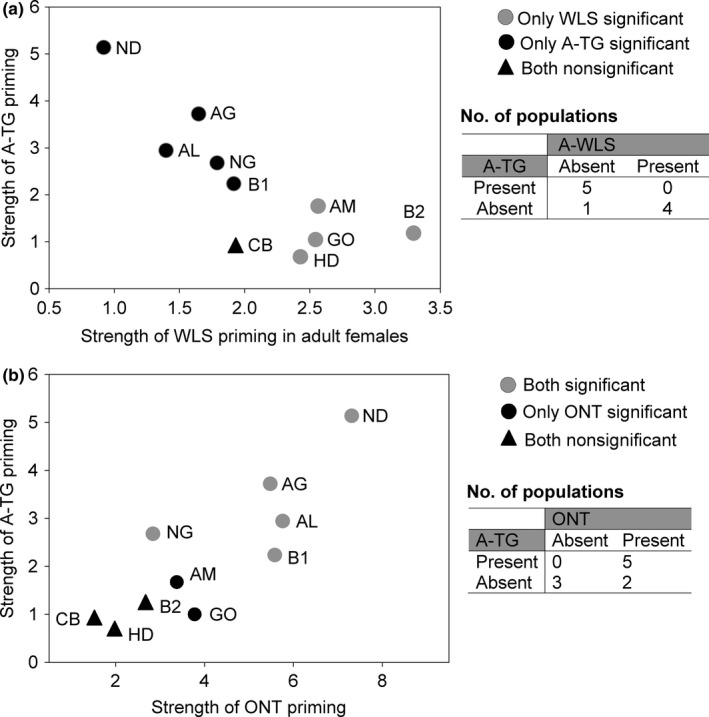
Associations between within‐generation and transgeneration immune priming. Strength of A‐TG response in male offspring as a function of (a) strength of WLS immune priming in female adults and (b) ONT priming in males. Strength of priming was estimated as described in Figure 3. Each population (labeled) was categorized based on the presence or absence of each type of priming response (using significant hazard ratios as explained in Figure 3), and contingency tables (shown beside each panel) were used to test the association between two types of immune priming across populations. WLS, Within‐life stage immune priming; A‐TG, transgenerational benefits of priming adult females, ONT, ontogenic immune priming

We tested whether the different types of immune priming responses were associated within populations. We found that most populations either showed significant female WLS priming or significant TG priming in male offspring, but not both (Figure 5a; Fisher's exact test, *p* = .046). In contrast, there was no association between female WLS and TG priming in female offspring (Fig. S18A; Fisher's exact test, *p* = .643). We also found a nonsignificant trend for an association between ONT priming in males and TG priming in male offspring (Figure 5b; Fisher's exact test, *p* = 0.167), but not for female offspring (Fig. S18B; Fisher's exact test, *p* = .663). For male offspring, one of the two populations that showed only ONT priming had nearly significant TG priming (population AM, Figure 5b; *p* = .059). If this population were counted as showing both types of priming, the association between ONT and male TG priming would be significant (Fisher's exact test, *p* = .046). Although the association is not strong, these results suggest that in populations where male adults benefit from larval ONT priming, they may also benefit from maternal TG immune priming. Overall, our results indicate that transgenerational immune priming responses are associated with within‐generation responses, but the association is limited to male offspring.

## 4 Discussion

Our work provides the first evidence of substantial variation in both within‐generation and transgenerational immune priming responses among natural populations of an insect. Approximately half the populations did not show a significant response to any given type of priming; on the other hand, all populations showed at least two forms of priming. Relative to unprimed controls, primed individuals showed up to 13‐fold higher survival in some cases, whereas others showed no benefits of priming. Note that we reared wild‐collected beetles under standard laboratory conditions for 9–10 generations before starting our experiments; hence, we probably underestimated the variation in priming responses across populations. What is the cause of this variability? Potential hypotheses include gain and loss of priming responses via genetic drift; local adaptation to specific pathogen diversity and abundance (Sutton, Nakagawa, Robertson, & Jamieson, 2011); variable life history‐related costs associated with immune investment (Miller, White, & Boots, 2006; Roy & Kirchner, 2000); and variable susceptibility to pathogens (Best, Tidbury, White, & Boots, 2013). Currently, we cannot directly test these hypotheses as we do not have information on the local pathogen pressure experienced by our beetle populations, the fitness costs of immune priming, or their relative susceptibility to *B. thuringiensis*. Nonetheless, our work demonstrates the importance of quantifying variability of immune priming responses in natural populations and sets up a framework to understand the evolution of immune priming responses.

One of our most interesting findings is that ontogenic priming confers a greater survival benefit than within‐life stage or transgenerational immune priming response. A recent theoretical model predicts that if adults incur higher costs of infection than larvae, selection should favor strong ontogenic priming that reduces the proportion of susceptible adults (Tate & Rudolf, 2012). On the other hand, transgenerational priming should be favored when larvae are more susceptible to infection than adults. Thus, if *B. thuringiensis* imposes stage‐specific costs of infection in *T. castaneum*, it may have selected for stronger ontogenic priming in our populations. In a separate experiment, we found that larvae and adults from a laboratory‐adapted, outcrossed flour beetle population were equally susceptible to *B. thuringiensis* infection (Fig. S19A). These data suggest that beetle life stages are not differentially susceptible to infection, although it is possible that our natural populations do show stage‐specific susceptibility. An alternative explanation is that larval priming inherently induces stronger and longer‐lasting immune responses that persist through metamorphosis and confer protection against subsequent infection in adulthood. For instance, blood cells that constitute insect cellular immunity differentiate primarily in larval lymph glands (Grigorian & Hartenstein, 2013; Waltzer, Gobert, Osman, & Haenlin, 2010) and play a major role in the priming response in mosquitoes (Rodrigues, Brayner, Alves, Dixit, & Barillas‐mury, 2012) and fruit flies (Pham et al., 2007). If priming is generally dependent on blood cell differentiation, it may explain our observation of relatively weaker adult priming responses. Another interesting result from our analysis is that the strength of larval TG priming is similar to the strength of adult TG priming, but much weaker than larval ontogenic priming. Thus, the high survival benefit of ONT priming (through metamorphosis) is not transmitted to the next generation. Hence, we speculate that during oviposition, priming is “reset,” perhaps because the mechanisms responsible for ontogenic and transgenerational priming are different. In honeybees (*Apis mellifera*), transgenerational priming is mediated via fragments of bacterial antigens that are transported to offspring via egg yolk proteins (Salmela, Amdam, & Freitak, 2015). However, no such mechanism has been clearly elucidated for ontogenic priming. Further empirical studies are thus critical to elucidate the complex interplay between immune priming types and their relative impact on the outcome of infection within a population.

Our data also revealed novel associations between within‐generation and transgenerational immune priming responses. In populations where adult females showed significant within‐life stage immune priming, male offspring did not show transgenerational priming. We speculate that this negative relationship may reflect a tradeoff between maternal and offspring immunity (Moreau et al., 2012): transferring immunity to offspring may be costly for females who also bear the cost of their own immune priming response. However, this needs to be explicitly tested by quantifying the difference in the priming response of offspring of individual females that were primed and challenged as adults, vs. females that were not primed and challenged. Our results also suggest a weak association between male ONT and male TG priming. Interestingly, both relationships between transgenerational and within‐generation priming were limited to male offspring. Such male‐specific associations may arise due to sex‐specific variation in infection susceptibility, investment in other immune components, or tradeoffs with other fitness components. We cannot test these predictions as the relative impact of *B. thuringiensis* infection in both sexes is unknown in natural beetle populations. However, separate experiments with an outbred *T. castaneum* population showed that infected males die about twice as fast as females (Fig. S19B). It is possible that the natural populations analyzed here also show similar sex‐specific variation in susceptibility to infection, and further work is necessary to distinguish between these hypotheses.

We suggest that our results are applicable in many insect‐pathogen systems. *B. thuringiensis* infects multiple insect hosts (Bravo, Likitvivatanavong, Gill, & Soberón, 2011) and is commonly found in diverse habitats such as soil, water, and grain dust (Argôlo‐filho & Loguercio, 2014; Lambert & Peferoen, 2014). Hence, *B. thuringiensis* may impose strong selection on many insects occupying diverse ecological niches, influencing the evolution of their immune responses in the wild. Although we did not test whether the specificity of the priming response varies across populations, we showed that larvae and adult females from an outcrossed population (established by mixing the natural populations used in this study) exhibited pathogen‐ and strain‐specific WLS priming responses. Beetles produced a priming response against *B. thuringiensis* only if they were previously primed with the same species (Fig. S20). Priming with another bacterial pathogen *Bacillus subtilis* failed to confer protection against *B. thuringiensis* infection. Larvae and adult females could even distinguish between strains of *B. thuringiensis*, showing a survival advantage following an infection only when they had been previously primed with the same strain of the bacterium (see Fig. S21). Another study by Roth and coworkers (Roth et al., 2009) demonstrated a similar strain‐specific WLS priming response against *B. thuringiensis* in *Tribolium* larvae of a different outcrossed population. Together, these results indicate that populations of diverse genetic background show a very specific within‐life stage priming response against the bacterial pathogen that we used in this study. Hence, we assume that the observed WLS immune priming response in natural populations is most likely a specific response against *B. thuringiensis* and does not represent general protection via an overall upregulation of immune components. Nevertheless, further work is necessary to test this assumption explicitly. Also, we do not know whether other forms or priming such as ONT and TG show such high specificity. We note that we assayed immune priming response using septic injury, whereas many pathogens infect their insect hosts via the oral route. However, recent studies confirm that both septic injury (Roth et al., 2009) and oral infection (Milutinović, Fritzlar, & Kurtz, 2014) with *B. thuringiensis* produce comparable immune priming responses in *Tribolium* beetles, suggesting that our infection protocol is unlikely to bias our results.

We would like to end by highlighting several open questions that have emerged from our work. (i) Do sex‐ and stage‐specific differences in immune function and pathogen susceptibility explain the observed variation in immune priming response? (ii) Do variable fitness costs of immune priming explain the observed variation in the priming response across populations? (iii) Finally, do mechanisms underlying various forms of immune priming differ from each other? We suggest that future work on insect immune priming should focus on variation in the mechanistic and ecological and evolutionary aspects of natural variation in immune priming. In particular, experimental manipulation of specific immune priming types across sexes and life stages promises to shed light on the complex problem of immune priming responses and their variable outcomes in natural populations.
