# Supplementary material for: Divergent immune priming responses across flour beetle life stages and populations
Source: Ecol Evol. 2016 Oct 9;6(21):7847–55. doi: 10.1002/ece3.2532 (PMC6093166; doi:10.1002/ece3.2532)
Supplement: Supplementary file 1 [file ECE3-6-7847-s001.docx]

**SUPPLEMENTARY INFORMATION**

**Supplementary table**

**Table S1.** Sample sizes across the treatments (Primed vs. unprimed) in each sex and population. LW = within life stage immune priming benefits in larvae, AW = within life stage immune priming benefits in adults, O = ontogenic priming; LT = trans-generational benefits of priming females at larval stage. AT = trans-generational benefits of priming adult females, F = Female, M = Male, N_P_ = Starting number of individuals for priming with heat-killed *B*. *thuringiensis* (or mock priming with insect Ringer), N_C_ = Final number of individuals for challenge with live *B*. *thuringiensis*, N_CF_ = Number of challenged female offspring, N_CM_ = Number of challenged male offspring. Groups where we found mortality between the period of priming and challenge are highlighted in bold.

|  |  | **LW** |  | **AW** |  |  |  | **O** |  |  |  | **LT** |  | **AT** | |  | |
| --- | --- | --- | --- | --- | --- | --- | --- | --- | --- | --- | --- | --- | --- | --- | --- | --- | --- |
|  |  |  |  | **F** |  | **M** |  | **F** |  | **M** |  |  |  |  |  | |  |
| **Population** | **Treatment** | **N_P_** | **N_C_** | **N_P_** | **N_C_** | **N_P_** | **N_C_** | **N_P_** | **N_C_** | **N_P_** | **N_C_** | **N_CF_** | **N_CM_** | **N_CF_** | **N_CM_** | |  |
| AG | Primed | 31 | 31 | 33 | 33 | 12 | 12 | 16 | 16 | 16 | 16 | 30 | 30 | 33 | 24 | |  |
| AL |  | 31 | 31 | 33 | 33 | 12 | 12 | 16 | 16 | 17 | 17 | 30 | 30 | 33 | 21 | |  |
| AM |  | 31 | 31 | 33 | 33 | 12 | 12 | 16 | 16 | 16 | 16 | 30 | 30 | 33 | 24 | |  |
| B1 |  | 31 | 31 | 33 | 33 | 12 | 12 | 16 | 16 | 16 | 16 | 30 | 30 | 17 | 20 | |  |
| B2 |  | 31 | 31 | 33 | 33 | 12 | 12 | 16 | 16 | 16 | 16 | 30 | 30 | 32 | 24 | |  |
| CB |  | 31 | 31 | 33 | 33 | 12 | 12 | 16 | 16 | 16 | 16 | 30 | 30 | 32 | 21 | |  |
| GO |  | 31 | 31 | 33 | 33 | 12 | 12 | **16** | **15** | 16 | 16 | 30 | 30 | 32 | 23 | |  |
| HD |  | 31 | 31 | 33 | 33 | 12 | 12 | 16 | 16 | 16 | 16 | 30 | 30 | 33 | 23 | |  |
| ND |  | 31 | 31 | 33 | 33 | 12 | 12 | **16** | **15** | **16** | **14** | 30 | 30 | 20 | 18 | |  |
| NG |  | 31 | 31 | 33 | 33 | 12 | 12 | **16** | **15** | 16 | 16 | 30 | 30 | 24 | 18 | |  |
| AG | Unprimed | 30 | 30 | 33 | 33 | 12 | 12 | 16 | 16 | 16 | 16 | 30 | 30 | 41 | 19 | |  |
| AL |  | 30 | 30 | 32 | 32 | 12 | 12 | 16 | 16 | 16 | 16 | 30 | 30 | 31 | 22 | |  |
| AM |  | 30 | 30 | 32 | 32 | 12 | 12 | 16 | 16 | 16 | 16 | 30 | 30 | 33 | 23 | |  |
| B1 |  | 30 | 30 | 32 | 32 | 12 | 12 | 16 | 16 | 16 | 16 | 30 | 30 | 25 | 26 | |  |
| B2 |  | 30 | 30 | 32 | 32 | 12 | 12 | 16 | 16 | 16 | 16 | 30 | 30 | 37 | 24 | |  |
| CB |  | 30 | 30 | 32 | 32 | 12 | 12 | 16 | 16 | **17** | **16** | 30 | 30 | 32 | 19 | |  |
| GO |  | 30 | 30 | 33 | 33 | 12 | 12 | 16 | 16 | 16 | 16 | 30 | 30 | 36 | 21 | |  |
| HD |  | 30 | 30 | **33** | **32** | 12 | 12 | **16** | **15** | 16 | 16 | 30 | 30 | 37 | 26 | |  |
| ND |  | 30 | 30 | 32 | 32 | 12 | 12 | 16 | 16 | 16 | 16 | 30 | 30 | 34 | 20 | |  |
| NG |  | 30 | 30 | **33** | **32** | 12 | 12 | **17** | **15** | **17** | **16** | 30 | 30 | 27 | 26 | |  |

**Supplementary figures**

**Figure S1.**

**Figure S1.** Collection sites of beetle populations from India. AG = Agartala, Tripura; AM = Ahmedabad, Gujarat; AL = Allahabad, Uttar Pradesh; B1 & B2 = 2 populations collected from Bangalore, Karnataka; ND = Nadia, West Bengal; CO = Coochbehar, West Bengal; HD = Hyderabad, Telangana; GO = Vasco, Goa; NG = Nagpur, Maharashtra.

**
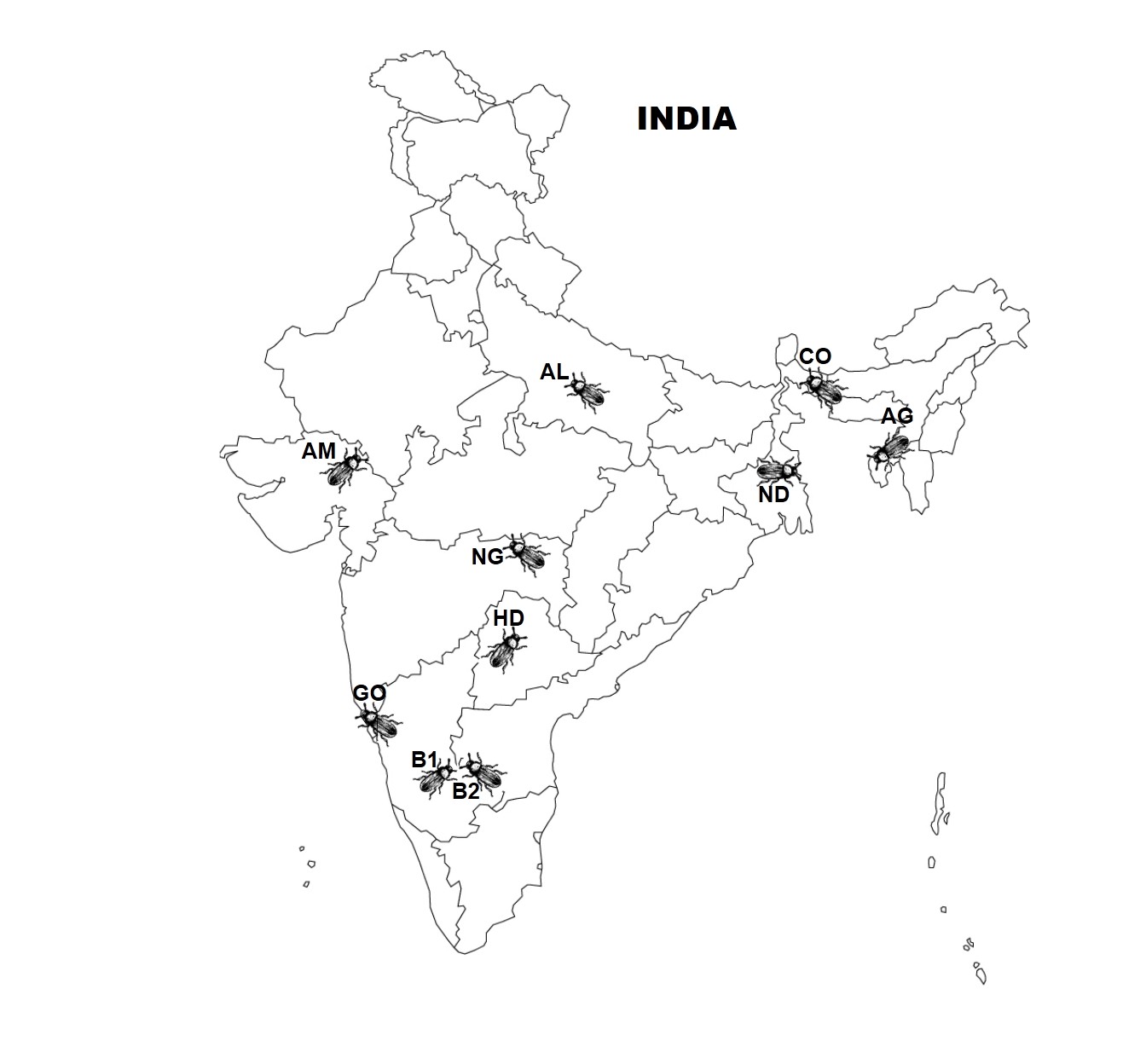
**

**Figure S2.** Development rate (i.e. days to pupation) of eggs from each population, measured in isolation in wheat flour. n = 20 eggs/population


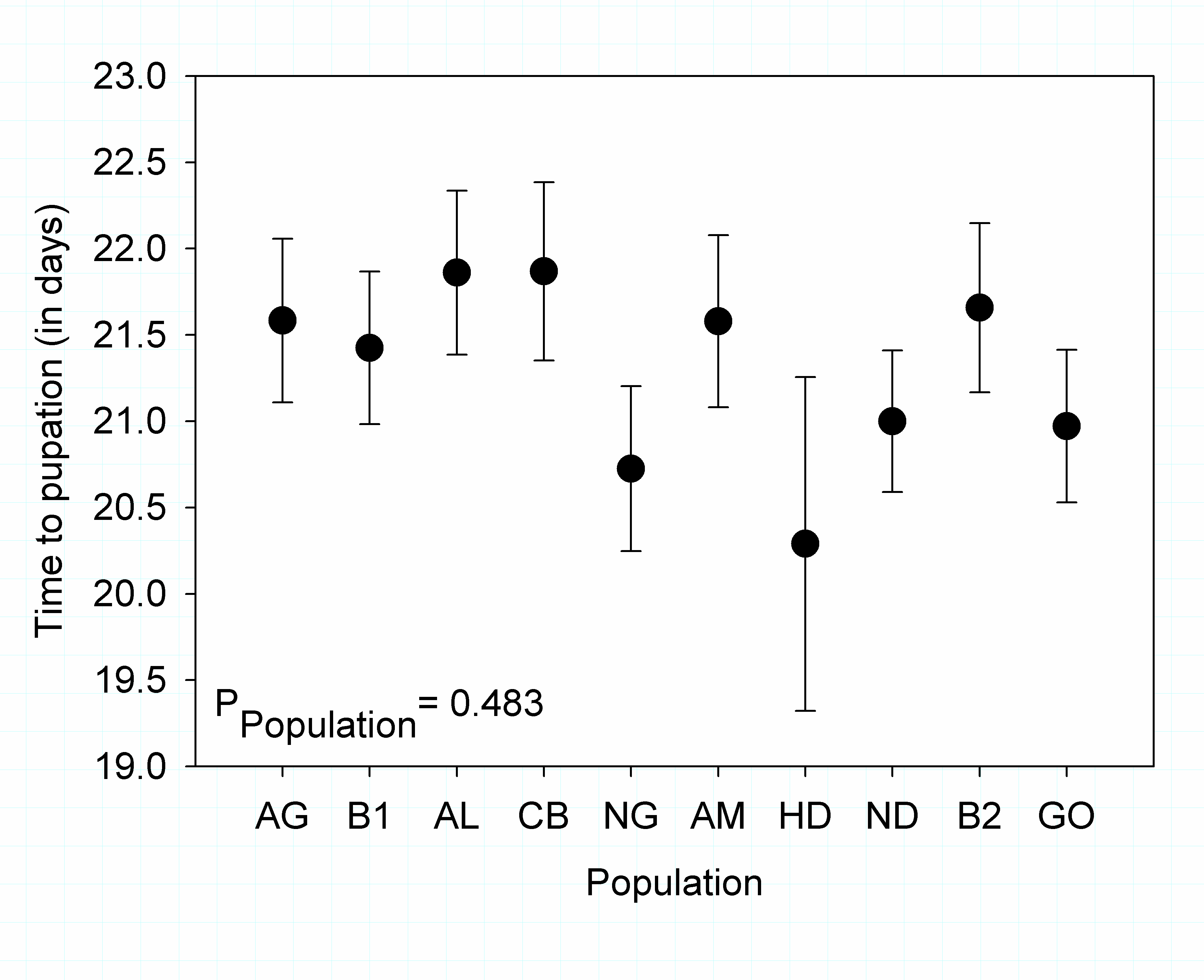


**Figure S3.** Frequency distribution of the number of offspring produced by control females for the A-TG priming assay. Values within parentheses in each panel indicate the percentage of females that produced less than 5 offspring (pupae) after 3 weeks of development, and were excluded from further analysis. The dashed line in each panel indicates the cut-off value of 5 offspring.





**Figure S4.** Frequency distribution of the number of offspring produced by primed females for the A-TG priming assay. Values within parentheses in each panel indicate the percentage of females that produced less than 5 offspring (pupae), and were excluded from further analysis. The dashed line in each panel indicates the cut-off value of 5 offspring.





**Figure S5.** Frequency distribution of the number of offspring produced by control females for the L-TG priming assay. Values within parentheses in each panel indicate percentage of females that produced less than 5 offspring (pupae), and were excluded from further analysis. The dashed line in each panel indicates the cut-off value of 5 offspring.





**Figure S6.** Frequency distribution of the number of offspring produced by primed females for the L-TG priming assay. Values within the parentheses in each panel indicate percentage of females that produced less than 5 offspring (pupae), and were excluded from further analysis. The dashed line in each panel indicates the cut-off value of 5 offspring.

**

**

**Figure S7.** Survival curves for larvae from each population, after within-life stage immune priming. P values for the impact of immune priming treatment are reported in each panel.





**Figure S8.** Survival curves for adult females from each population, after within life stage immune priming. P values for the impact of immune priming treatment are reported in each panel.

**

**

**Figure S9.** Survival curves for adult males from each population, after within life stage immune priming. P values for the impact of immune priming treatment are reported in each panel.





**Figure S10.** Survival curves for adult females from each population, after ontogenic priming. P values for the impact of immune priming treatment are reported in each panel.





**Figure S11.** Survival curves for adult males from each population, after ontogenic priming. P values for the impact of immune priming treatment are reported in each panel.





**Figure S12.** Survival curves for adult females from each population, after trans-generational benefits of priming adult females. P values for the impact of immune priming treatment are reported in each panel.





**Figure S13.** Survival curves for adult males from each population, after trans-generational benefits of priming adult females**.** P values for the impact of immune priming treatment are reported in each panel.


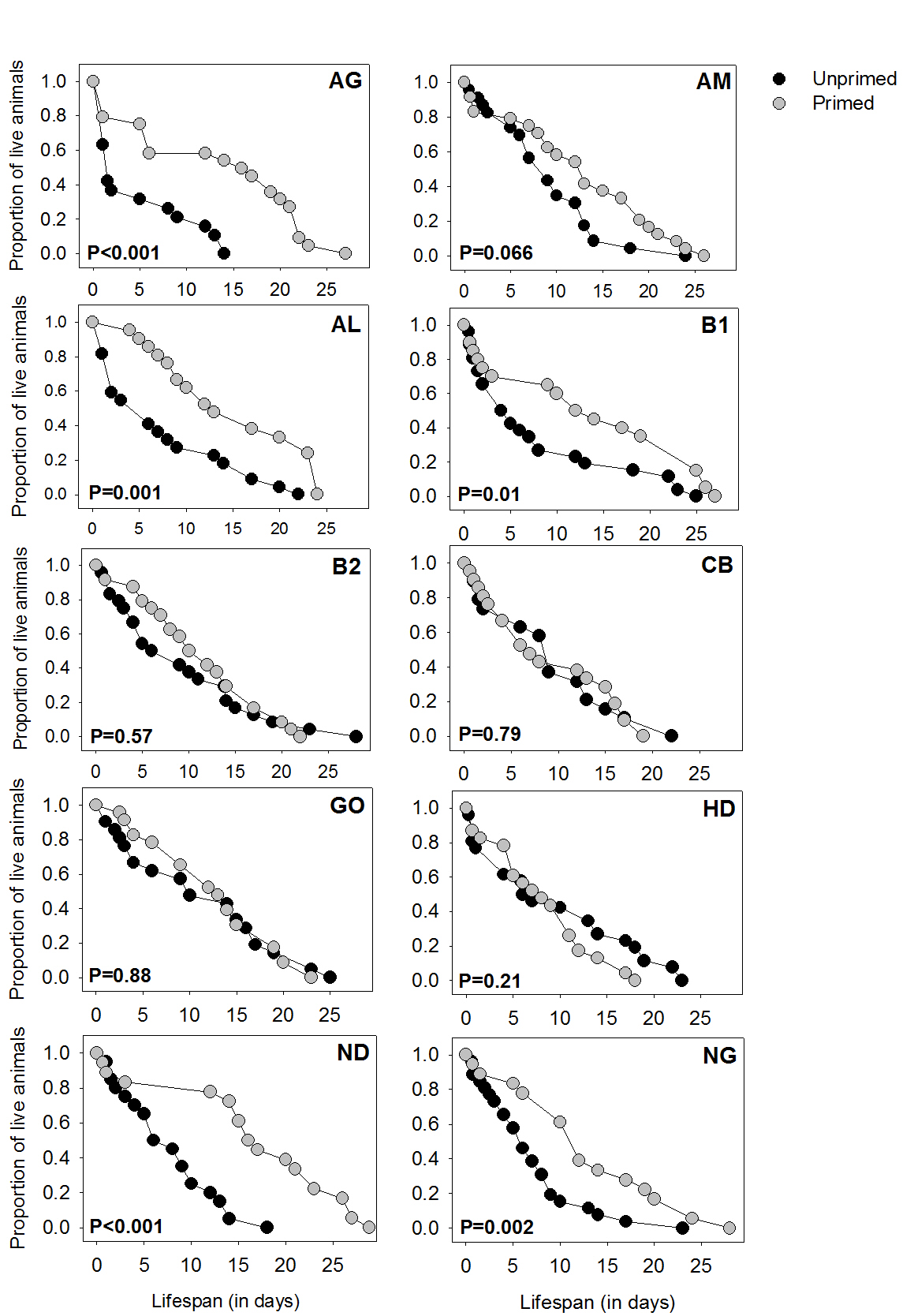


**Figure S14.** Survival curves for adult females from each population, after trans-generational benefits of priming females at larval stage. P values for the impact of immune priming treatment are reported in each panel.

**

**

**Figure S15.** Survival curves for adult males from each population, after trans-generational benefits of priming females at larval stage. P values for the impact of immune priming treatment are reported in each panel.

**

**

**Figure S16.** (A) Comparison between hazard ratios (strength of priming) estimated from the full dataset vs. a truncated data set (n=17 females/treatment/population, sampled 5 times with replacement). (B-C) Impact of sample size (n=15 to 30 females /treatment/population) on estimated hazard ratios for two populations: (B) Population AG, which did not show significant A-TG priming, and (C) Population GO, which did show significant A-TG priming response with the full dataset. Error bars corresponds to 5 subsampling iterations.





**Figure S17.** Comparing hazard ratios (strength of priming) between the original ONT priming assay and the repeat assay (grey circles = populations that showed significant ONT priming in the original assay as well as in the repeat assay; black circles = populations that did not show a benefit of ONT priming in both assays).





**Figure S18.** Association between within- and trans-generation immune priming. Strength of trans-generational adult (maternal) immune priming (A-TG) in female offspring as a function of the strength of (A) within life stage immune priming (WLS) in female adults and (B) ontogenic priming (ONT) in females. Strength of priming was estimated as described in Figure 3 (see the main text). Each population (labelled) was categorized based on the presence (significant: p ≤ 0.05) or absence (nonsignificant: p > 0.05) of each type of priming response and contingency tables (shown beside each panel) were generated to test the associations between two types of immune priming across populations.





**Figure 19**. (A) Stage- and (B) Sex-specific impact of *B. thuringiensis* infection in a laboratory adapted outbred population.

**

**

**Figure S20.** Pathogen-specific WLS priming response in (A) larvae and (B) adult females. Priming was performed with heat killed bacterial pathogens *Bacillus thuringiensis* (Bt1; DSM. No. 2046) or *Bacillus subtilis* (Bs; MTCC 2451), followed by a subsequent challenge with live Bt1 (see the main text for methods). Control treatments: Control = mock priming followed by a mock challenge, UP-Bt1= Mock priming followed by a challenge with Bt1, UP-Bs= Mock priming followed by a challenge with Bs; Homologous priming-challenge combination: Bt1-Bt1= Primed and challenged with Bt1; Heterologous priming-challenge combination: Bs-Bt1= Primed with Bs followed by a challenge with Bt1. Only the homologous priming-challenge combinations produced strong priming response (Proportional hazard test; p<0.01 for each pairwise comparison between unprimed control and primed individuals), whereas beetles that received heterologous priming-challenge combinations did not show a survival advantage.

**

**

**Figure S21.** Strain-specific WLS priming response in (A) larvae and (B) adult females. Two strains of *B. thuringiensis*, DSM. No. 2046 (Bt1, used in this study) and MTCC 6905 (Bt2, additional strain) were used for priming. Primed individuals were subsequently challenged with live Bt1 (see main text for detailed methods). Control treatments: Control = mock priming followed by a mock challenge, UP-Bt1= Mock priming followed by a challenge with Bt1, UP-Bt2= Mock priming followed by a challenge with Bt2; Homologous priming-challenge combination: Bt1-Bt1= Primed and challenged with Bt1; Heterologous priming-challenge combination: Bt2-Bt1= Primed with Bt2 followed by a challenge with Bt1. Only homologous priming-challenge combinations produced a strong priming response (Proportional hazard test; p<0.01 for each pairwise comparison between unprimed control and primed individuals), whereas heterologous combinations did not show a survival benefit.

**

**
